# Cardiac Monitoring for Patients Undergoing Treatment With MEK Inhibitor Monotherapy

**DOI:** 10.1007/s11912-026-01814-2

**Published:** 2026-08-21

**Authors:** Jondavid Menteer, Devorah Segal, Stefania Maraka, Laura J. Klesse, Prakash Ambady, Michael G. Fradley

**Affiliations:** 1 Keck School of Medicine, Children’s Hospital Los Angeles, University of Southern California, Los Angeles, CA USA; 2 New York University Langone Health, NYU Grossman School of Medicine, New York, NY USA; 3 University of Illinois at Chicago, Chicago, IL USA; 4 University of Texas Southwestern Medical Center/Children’s Health, Dallas, TX USA; 5 Providence Brain & Spine Institute, Portland, OR USA; 6 Hospital of the University of Pennsylvania, Philadelphia, PA USA; 7 Division of Cardiology, Children’s Hospital Los Angeles, 4650 W Sunset Blvd, Los Angeles, CA 90027 USA

**Keywords:** MEK inhibitors, Cardiac monitoring, Cardiotoxicity, Echocardiography, Cardiac biomarkers, NF1

## Abstract

**Purpose of Review:**

Aberrant activation of the RAS-RAF-MEK-ERK signaling pathway contributes to numerous malignancies, congenital syndromes, and cardiovascular disorders, establishing MEK inhibitors (MEKi) as crucial therapeutic agents. MEKi demonstrate substantial clinical benefit as monotherapy (e.g., NF1-associated tumors) or combination therapy (e.g., melanoma, glioma, congenital RASopathies). Cardiovascular adverse events, primarily asymptomatic reductions in left ventricular ejection fraction (LVEF), have prompted intensive echocardiographic monitoring.

**Recent Findings:**

Emerging evidence indicates that isolated mild LVEF decreases rarely progress to symptomatic heart failure, raising concerns about unnecessary treatment interruptions driven by overly rigorous imaging protocols.

**Summary:**

Integrating cardiac biomarkers into monitoring strategies could offer a more pragmatic approach, guiding echocardiographic evaluations based on biomarker elevation or symptoms. Expert recommendations advocate personalized, risk-adapted surveillance frameworks. High-risk patients include those with baseline cardiovascular conditions, abnormal biomarker profiles, or significant clinical symptoms. Normal-risk patients can be safely monitored clinically, reserving imaging for biomarker changes or symptom onset. Prospective studies should seek to validate these recommendations.

## Introduction

**Fig. 1:**
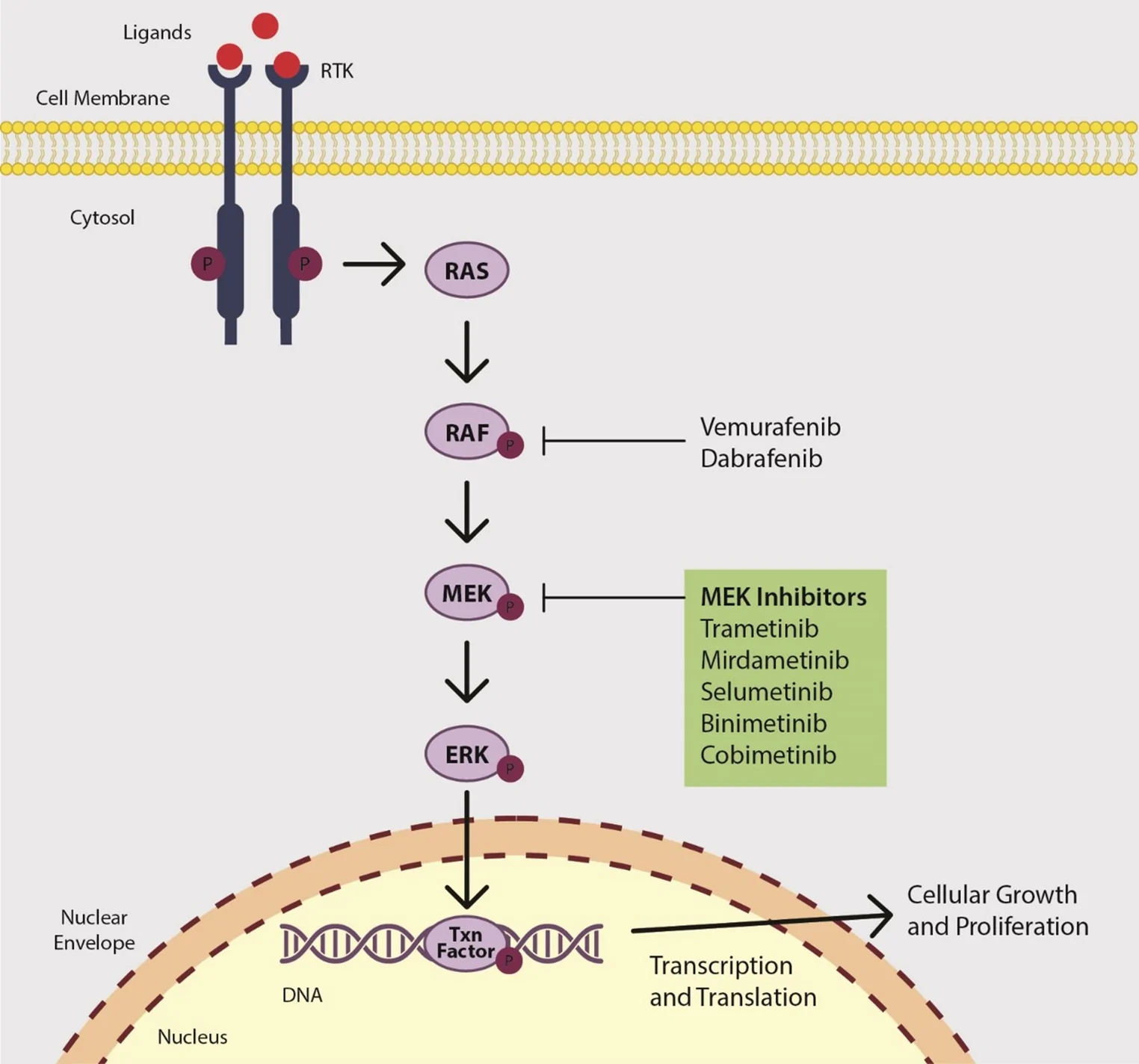
Intersection of RAS Pathway and MEKi [1]. Ligand binding to RTKs initiates the RAS-RAF-MEK-ERK signaling cascade, resulting in the transcriptional regulation of genes that control cellular proliferation and growth. MEKi specifically target MEK1/2 kinase activity to disrupt aberrant signaling in RAS-driven cancers and syndromes

The RAS-RAF-MEK-ERK signaling cascade, a central component of the mitogen-activated protein kinase (MAPK) pathway, plays a fundamental role in regulating cellular proliferation, differentiation, survival, and apoptosis (Fig. 1). Aberrations in this pathway are implicated in a wide range of both malignant and nonmalignant medical conditions, underscoring its broad pathophysiological relevance.

Activating mutations in *RAS* genes, particularly *KRAS*, lead to constitutive downstream signaling independent of upstream cues, driving sustained cellular proliferation and resistance to apoptosis. Similarly, *BRAF* mutations, especially the V600E substitution, result in autonomous kinase activity and are strongly associated with progression of certain melanomas, thyroid cancers, and gliomas. In these contexts, aberrant MAPK pathway activation serves as both a driver of tumorigenesis and a predictive biomarker for targeted therapy [2–4]. These activating mutations are among the most common oncogenic events, occurring in roughly one‑quarter of all human cancers and reaching strikingly high frequencies in specific tumor types—approximately 90% of pancreatic ductal adenocarcinomas, 50% of colorectal and thyroid cancers, 30% of lung adenocarcinomas, and 25% of melanomas.

Beyond sporadic cancers, germline pathogenic variants affecting this signaling axis are characteristic of several systemic disorders. Neurofibromatosis type 1 (NF1) is caused by loss-of-function variants in the *NF1* gene, which encodes neurofibromin—a tumor suppressor that negatively regulates RAS activity [5]. Individuals with NF1 exhibit augmented MAPK signaling, predisposing them to a spectrum of benign and malignant tumors, including neurofibromas (both cutaneous and plexiform), gliomas (most commonly of the optic nerve), and malignant peripheral nerve sheath tumors (MPNSTs) [5–7]. Given this prevalence, MAPK kinase inhibitors (MEKi)—already central to NF1‑associated tumor therapy—have broad potential across RAS‑driven malignancies, making careful evaluation of their cardiac‑toxicity profile increasingly important as their use expands [4]. Other RASopathies, such as Noonan, Costello, and cardio-facio-cutaneous syndromes, result from germline activating mutations in *RAS*, *RAF*, *MEK*, or *ERK* genes, and they present with overlapping phenotypes, including facial dysmorphia, congenital heart defects (CHDs), lymphangiectasia, and hypertrophic cardiomyopathy (HCM) [8–10].

In cardiovascular physiology, the MEK-ERK arm of the MAPK pathway mediates adaptive responses, such as cardiomyocyte hypertrophy, angiogenesis, and endothelial function. Disruption of this finely tuned signaling—whether by genetic defects or pharmacologic inhibitors—can lead to pathologic cardiac remodeling and increase vulnerability to injury.

Collectively, these insights underscore the RAS-RAF-MEK-ERK pathway as a critical nexus in human disease, with implications that extend across oncology, developmental disorders, and cardiovascular health. Therapeutic strategies targeting this cascade—particularly MEKi—are becoming more widely utilized, either alone or as combination drug therapy for the aforementioned conditions, and must therefore be balanced against the pathway’s integral role in normal tissue physiology.

One particular area of increased MEKi use is in patients with NF1-associated plexiform neurofibromas (NF1-PNs). The first MEKi was approved in 2020 by the US Food and Drug Administration (FDA) for pediatric patients with NF1-PN who have PNs that are symptomatic and not amenable to complete resection, with a second drug approved in 2025 for both pediatric and adult patients. NF1 is an autosomal dominant disorder that affects approximately 1 in 2500 to 3000 individuals worldwide, regardless of sex or ethnic background [11, 12]. Among the various tumors associated with NF1, PNs are particularly significant. PNs are complex, often congenital tumors that arise from multiple nerve fascicles and can infiltrate surrounding tissues. They are estimated to occur in approximately 30% to 50% of individuals with NF1 [13–15]. PNs can cause substantial morbidity, including pain, disfigurement, functional impairment, and even life-threatening complications if they compress vital structures. Importantly, PNs can transform into MPNSTs over a patient’s lifetime [7, 13].

Historically, surgical resection has been the primary treatment for symptomatic PNs; however, complete excision is often difficult due to their diffuse nature, including infiltration into the nerve and adjacent structures. Development of PNs in NF1 is due to overactivation of the RAS-MAPK pathway, which presents a unique opportunity for targeted medical therapy.

### Pharmacotherapy and the RAS-RAF-MEK-ERK Pathway

The RAS-RAF-MEK-ERK axis is initiated by ligand binding to membrane-bound receptor tyrosine kinases, which leads to the activation of RAS, followed by sequential phosphorylation of RAF kinases (CRAF-1, ARAF, BRAF), MEK1/2, and extracellular signal-regulated kinases (ERK1/2) [16–20]. Activated ERK proteins phosphorylate a wide range of substrates, including transcription factors, cytokines, and growth factor receptors, influencing key cellular functions such as angiogenesis, cell cycle progression (proliferation, differentiation), and inhibition of apoptosis [3, 21–23].

Prior to the mid-2010s, the direct pharmacological inhibition of RAS was limited by the protein’s lack of well-defined ligand targets [23]. Consequently, therapeutic strategies focused on interrupting the downstream signaling cascade, particularly the RAF-MEK-ERK axis [24, 25]. BRAF inhibitors were the first targeted therapeutics on this pathway, pre-dating the less selective pan-RAF inhibitors, but single-agent therapy frequently leads to the emergence of compensatory (reflexive) reactivation of the MAPK pathway [26, 27]. Combination strategies targeting both RAF and MEK have shown greater efficacy in overcoming resistance, though associated toxicities (including cardiac dysfunction) related to paralog redundancy in the MAPK pathway can limit the usefulness of this therapeutic approach [28–33].

More recently, MEKi—such as trametinib, cobimetinib, mirdametinib, selumetinib, and binimetinib—targeting the kinase activity of MEK1 and/or MEK2 have been used off-label as single agents (monotherapy) and have emerged as critical tools for disrupting aberrant MAPK signaling (Table 1) [34, 35]. These agents have demonstrated clinical benefit in cancers characterized by MAPK hyperactivation (e.g., gliomas, melanoma, and non–small cell lung cancer) [36–38], tumors driven by neurofibromin loss rather than direct *RAS* or *BRAF* mutations, and congenital RAS‑activation syndromes that feature lymphangiectasia and HCM.

**Table 1:** On-label and off-label uses of MEKi

MEKi	On-Label Indications	Off-Label Uses
Binimetinib	– Approved in combination with encorafenib for unresectable or metastatic melanoma with *BRAF* V600E/K mutations	Investigated in the following settings: – AML, biliary tract cancer, high-grade glioma – Colorectal cancer, NSCLC, including *KRAS*-mutant subtypes – *NRAS*-mutant tumors, ovarian cancer, brain metastases – Studied in combination with targeted agents such as buparlisib, erlotinib, and hydroxychloroquine–NF1-related PN (ongoing, phase II)
Cobimetinib	– Approved in combination with vemurafenib for unresectable or metastatic melanoma with *BRAF* V600E/K mutations – Approved as monotherapy for histiocytic neoplasms	Investigated in the following settings: – AML and multiple myeloma, often in combination with venetoclax or immune checkpoint inhibitors – Biliary tract cancer, colorectal cancer, and breast cancer – Craniopharyngiomas and glial tumors with *BRAF* V600 mutations – ALK-rearranged lung cancer, ovarian cancer, and anaplastic thyroid carcinoma
Mirdametinib	– Approved for NF1-related PN in adults and children aged 2 years or older	Studied in the following settings: – NF1-related symptomatic PN not amenable to complete resection in adults and children aged 2 years and older – Breast cancer, colorectal cancer, melanoma, and NSCLC – Low-grade glioma – Evaluated in combination with pan-HER inhibitors and PI3K/mTOR inhibitors
Selumetinib	– Approved for symptomatic, inoperable NF1-related PN in pediatric patients	Explored in the following settings: – Low- and high-grade gliomas, including both NF and non-NF1associated gliomas – Uveal melanoma, often in combination with dacarbazine – Metastatic breast cancer, NSCLC, pancreatic cancer, and thyroid cancer – Soft-tissue sarcomas
Trametinib	– Approved as monotherapy for *BRAF* V600E/K-mutant unresectable or metastatic melanoma – Approved in combination with dabrafenib for *BRAF*-mutant malignancies, including: • Adjuvant treatment of melanoma • NSCLC • Anaplastic thyroid cancer • LGG in pediatric patients – Approved in combination with dabrafenib under the FDA’s accelerated approval pathway for *BRAF* V600E-mutant solid tumors in patients without satisfactory treatment alternatives	Evaluated in the following settings: – Triple-negative breast cancer (with AKT inhibitors) – CNS tumors: high-grade gliomas, brain metastases – Colorectal cancer (with PD-1 blockade, EGFR inhibitors, chemotherapy) – Endometrial, hepatocellular carcinoma, epithelioid hemangioendothelioma – Leukemia, lymphoma, ovarian cancer – Rare *BRAF*-mutant cancers (ROAR trial): anaplastic thyroid carcinoma, biliary tract cancer - NF1-related PN and pediatric low-grade glioma (ongoing, Phase II)

ALK, anaplastic lymphoma kinase; AML, acute myeloid leukemia; CNS, central nervous system; EGFR, epidermal growth factor receptor; FDA, US Food and Drug Administration; LGG, low-grade glioma; MEKi, mitogen-activated protein kinase inhibitor; NF1, neurofibromatosis type 1; NSCLC, non–small-cell lung cancer; PD-1, programmed cell death protein 1; PN, plexiform neurofibroma

Loss of functional neurofibromin in NF1 amplifies RAS-MEK/ERK signaling, making MEK inhibition a rational and targeted therapeutic strategy [26, 27, 39]. MEKi, specifically mirdametinib and selumetinib, have demonstrated significant activity against inoperable PNs in patients with NF1, offering tumor shrinkage, symptom relief, and improved health-related quality of life [14, 15, 40]. MEKi have also demonstrated efficacy when utilized for progressive, NF1-associated, low-grade gliomas [41].

Elevated ERK activity correlates with disease severity in HCM, and syndromes with constitutive RAS/MAPK activation (e.g., Noonan, Leopard) frequently present with hypertrophic cardiac phenotypes [8, 42]. In Noonan syndrome and other congenital disease states involving constitutive activation of the RAS pathway, infancy can be particularly treacherous. HCM can be a morbid or mortal condition in young patients with these syndromes with no other disease-modifying treatments, and lymphangiectasia can cause life-threatening respiratory failure, intestinal failure, heart failure, effusions (pleural, pericardial, ascitic), and more. MEKi have been applied in these disease states with some success but without prospective study thus far [43]. Infants with Noonan‑associated HCM and heart failure face a 22% mortality rate within the first year, underscoring the need for effective therapies [44]. MEKi treatment has gained favor among pediatric cardiology and heart failure specialists, leading to the development of harmonization protocols to treat such morbid and mortal issues in young children [45]. Accordingly, the Advanced Cardiac Therapies Improving Outcomes Network now provides MEKi dosing and monitoring guidance that hinges on serial echocardiography and laboratory testing, with clinically meaningful endpoints focused on overt heart failure outcomes rather than symptomatic left ventricular ejection fraction (LVEF) fluctuations.

As MEKi therapies are used more broadly, there is a need to rationalize and adjudicate the inconvenient and expensive cardiac monitoring that has been perpetuated by pharmacovigilance in Phase 2 and 3 trials. Indeed, the amount of cardiac monitoring currently advocated by package inserts and pharmacovigilance protocols potentially reduces the number of patients with access to these effective therapies in centers where access to such cardiac monitoring is impaired (for cost or infrastructure reasons) and may reduce the effectiveness of therapy by provoking needless drug interruptions.

### Cardiovascular Toxicities Associated With MEKi

The MEK-ERK pathway is central to cardiac physiology, mediating cardiomyocyte hypertrophy, myocardial remodeling, and cellular survival. Deletion of CRAF‑1 or ERK2 in animal models markedly increases baseline and stress‑induced myocardial apoptosis, while downstream protection appears to be partly mediated by interleukin‑10–driven suppression of tumor necrosis factor‑α apoptosis signaling and by ATP‑sensitive potassium channel stabilization [9, 46–48]. Against this background, inhibition of MEK predictably lowers the myocardium’s threshold for injury and compromises recovery from insults such as pressure overload or ischemia‑reperfusion.

Across clinical experience, a reversible decline in LVEF emerges as the most clinically significant cardiovascular adverse event (CVAE) with MEKi. A meta‑analysis of 5 melanoma trials involving 2317 participants showed that combined BRAF and MEK blockade produced an 8.1% incidence of LVEF decline, versus 2.0% with BRAF inhibition alone (relative risk, 3.72) [49]. Concordant findings appear in individual studies: 7% of trametinib-treated patients in METRIC and 4% of patients receiving dabrafenib + trametinib versus 2% of those on dabrafenib alone in COMBI-d experienced LVEF-related events [29, 50].

In monotherapy settings, MEKi have demonstrated a consistent, though modest, incidence of LVEF decline. Trametinib monotherapy was associated with LVEF reduction in 7% of patients in early trials [29, 51]. In the ReNeu trial, mirdametinib monotherapy for NF1-PN resulted in treatment-related LVEF reduction (any Common Terminology Criteria for Adverse Events [CTCAE] v.5.0 grade) in 12% of adults and 20% of children, with no adults and only 1 child (2%) meeting CTCAE grade 3 criteria (LVEF < 40% or ≥ 20% decreased from baseline; Table 2). No adult or pediatric patients suffered symptomatic cardiac dysfunction/heart failure meeting CTCAE criteria (Table 2). Of the 15 pediatric patients reporting asymptomatic ejection fraction decreases, only 1 patient interrupted treatment [15].

**Table 2:** CTCAE v.5.0 cardiac adverse event grading criteria

CTCAE Term	Grade 1	Grade 2	Grade 3	Grade 4	Grade 5
EF decreased	—	Resting EF 50 - 40%; 10 - 19% drop from baseline	Resting EF 39 - 20%; ≥20% drop from baseline	Resting EF <20%	-
Heart failure	Asymptomatic with laboratory (e.g., BNP) or cardiac imaging abnormalities	Symptoms with moderate activity or exertion	Symptoms at rest or with minimal activity or exertion; hospitalization; new onset of symptoms	Life‑threatening consequences; urgent intervention indicated (e.g., continuous IV therapy or mechanical hemodynamic support)	Death
Left ventricular systolic dysfunction	—	—	Symptomatic from EF drop; responds to intervention	Refractory/poorly controlled HF due to EF drop; advanced interventions (e.g., MCS, transplant) indicated	Death

CTCAE, Common Terminology Criteria for Adverse Events; EF, ejection fraction; IV, intravenous; MCS, mechanical circulatory support

In pediatric cohorts, mild or borderline dysfunction with LVEF 45% to 50% is frequently observed but rarely, if ever, symptomatic [14, 52]. Robison et al. reported on a group of pediatric patients treated with MEKi at a large center and found a high incidence (50%) of borderline to mild, self-limited, variable LV dysfunction that never progressed to clinical heart failure [52].

MEKi in combination with BRAF inhibitors are used in certain patients with *BRAF*-mutant melanoma and other disease states. This combination therapy can cause a distinct pattern of CVAEs and toxicities beyond those seen with MEKi therapy alone. This manuscript focuses on MEKi monotherapy, which represents a distinct therapeutic avenue for certain disease states where co-targeting RAF is either unnecessary or inappropriate.

It should be noted that the monitoring advice provided in this manuscript should not be applied to patients on combination therapy with MEKi + BRAF inhibitors. That said, even pharmacovigilance data regarding combination therapy with MEKi + BRAF inhibition reinforce the predominance of asymptomatic LVEF decline, reporting incidence of roughly 3% and an odds ratio of 8.42 for BRAF + MEKi combinations, while symptomatic heart failure remains uncommon at roughly 3% even with the elevated risk of these combination therapies [53, 54]. Intensified surveillance protocols introduced in COMBI‑d/‑v, coBRIM, and COLUMBUS—baseline echocardiography followed by repeat imaging at week 4 and every 12 weeks—have heightened detection of subclinical dysfunction, though heterogeneity in the CTCAE classification of events (particularly LVEF declines within the normal range and LVEF declines to borderline values) continues to obscure precise incidence and trajectory of the more important cardiac changes [50, 55–57].

Centers with greater experience with MEKi therapy have grown to appreciate that clinically significant LVEF reduction is rare, and overt heart failure is an even more rare phenomenon in patients treated with MEKi monotherapy. In contrast, LVEF decreases tend to be asymptomatic and not a clinically meaningful variable (sometimes resolving even with continued therapy), and reversible with or without medication adjustment or discontinuation. This is an important observation because clinically silent LV dysfunction can only be detected by imaging studies, and if clinicians feel obliged to monitor for minor cardiac issues, any barrier to obtaining repeated echocardiographic studies (due to resource availability, cost, authorization, or reimbursement issues) may cause hesitation to utilize these effective medications, reducing the potential for patient benefit [15]. Further, one needs to consider the added opportunity costs of patients undergoing these tests, as they miss work, family time, and more, and divert precious hospital resources for this testing.

Other cardiovascular effects are less frequent and generally manageable. Arterial hypertension shows a modest increase with BRAF + MEK therapy compared with BRAF monotherapy (19.5% vs. 14.0%), an observation not corroborated by mixed‑method pharmacovigilance analyses [49, 53]. QT‑interval prolongation is rarely linked to MEKi monotherapy [29] but becomes more salient in combination regimens, with an adjusted reporting odds ratio of 6.13 for torsade de pointes/QT prolongation [53]. Asymptomatic creatine‑kinase elevations (not cardiac-specific CK-MB fraction), a recognized class effect of MEKi + BRAF therapy, occurred in up to 30% of cobimetinib‑treated patients in coBRIM, with grade ≥ 3 events in 12%, yet the elevations were typically controllable with dose modification [55, 58]. Syncope and supraventricular arrhythmias are infrequent but over‑represented in real‑world databases [53], while pericarditis has not been associated with MEKi [33, 59].

Taken together, these findings underscore the importance of structured cardiac surveillance and symptom-driven evaluation in patients treated with MEKi, in order to optimize the benefit-risk profile of MEKi-based therapies [29, 33, 49, 50, 53, 55, 57–62].

### MEK-ERK Signaling in Hypertrophy, Fibrosis, Contractility, Apoptosis, and LV Function

While reviewing the clinical impact of these pharmacologic agents on cardiac function, it is worthwhile to not only review the observed clinical experience, but also the underlying physiology regarding how MEK inhibition may impact cardiac contractility.

Several nonclinical studies have demonstrated that the MEK-ERK signaling pathway contributes to the cardiac hypertrophic response following stimulation or pressure overload. In cultured cardiomyocytes, MEK1-ERK1/2 signaling is activated by agonist stimulation and cell stretching and has been shown to promote hypertrophic gene expression and sarcomeric organization. Constitutive MEK1 activation enhances atrial natriuretic factor promoter activity, whereas inhibition via PD98059 (an experimental MEKi) or antisense oligonucleotides attenuates these effects, indicating that MEK/ERK signaling is necessary for hypertrophic remodeling in vitro [63–66]. The MEK-ERK pathway is also essential for cardiac adaptation to stress by preventing cardiomyocyte death. In cultured myocytes, growth factors and MAPK-activating agents protect against apoptosis induced by serum starvation, hypoxia, or reoxygenation. This protection is lost when MEK or ERK activity is pharmacologically inhibited [23, 63, 67].

Despite these findings, the role of MEK-ERK in hypertrophy continues to be debated. Some studies found no essential role for ERK in hypertrophy, and others suggested ERK activation may counteract hypertrophy under certain conditions [63, 68].

Beyond hypertrophy, MEK-ERK signaling has been implicated in interstitial fibrosis, contractility, and apoptosis. The p38 MAPK branch of the MAPK family—though distinct from MEK-ERK—has been shown to drive extracellular matrix remodeling and restrictive cardiomyopathy when chronically activated, without causing hypertrophy [69]. This indicates that dysregulated MAPK activity, more broadly, contributes to fibrosis and contractile dysfunction.

Regarding cardiomyocyte apoptosis, both experimental and genetic studies support a protective role for MEK-ERK signaling. In vivo, overexpression of the ERK-specific phosphatase DUSP6 inhibited ERK1/2 activity and predisposed hearts to increased apoptosis and heart failure after pressure overload, despite not altering hypertrophy itself [70]. This outcome is consistent with earlier findings that MEK-ERK protects cardiomyocytes from oxidative and ischemic injury [47]. Although the precise mechanisms are not fully understood, several downstream effectors of the RAS-RAF-MEK-ERK pathway also contribute to its antiapoptotic effects. These include mitochondrial regulation via BAD, Bcl-2, XIAP, MST2, and VDAC, as well as modulation of caspase 9, PKCε, and p53 [23, 47]. These findings demonstrate that inhibition of the MEK-ERK cascade impairs the heart’s ability to survive stress, highlighting the need for cardiac monitoring of some form during the use of therapies targeting this pathway.

Furthermore, MEK-ERK signaling does appear mechanistically important to maintaining LV systolic contractility. In ERK1/2-deficient models (Erk1^−/−^ and Erk2^+/−^), pressure overload led to greater cardiac decompensation despite preserved hypertrophy, underscoring the role of ERK1/2 in preserving functional reserve under stress [70]. Clinical findings in children treated long term with the MEKi selumetinib also support the importance of MEK-ERK in cardiac homeostasis. Although generally well tolerated, several patients developed asymptomatic decreases in LVEF after years of treatment, suggesting the need for ongoing cardiac monitoring and pointing to the role of MEK-ERK in sustaining cardiac function [52].

Mice with genetic inactivation of ERK1 or partial loss of ERK2 exhibit normal baseline cardiac size and function but show increased susceptibility to myocardial injury. Following ischemia-reperfusion, Erk2^+/−^ mice demonstrate greater infarct size, increased DNA fragmentation, and higher levels of TUNEL-positive (apoptotic or DNA fragmented) cardiomyocytes compared with controls. In contrast, mice with activated cardiac MEK1-ERK1/2 signaling are protected against apoptosis and maintain better hemodynamic function [47].

These findings suggest that ERK1/2 signaling is not essential for normal heart development or function under baseline conditions but becomes critical during stress. Therefore, the risk of MEKi-associated cardiotoxicity may be context-dependent, requiring an additional cardiac insult—such as ischemia, hypertension, shock, or perhaps other stressful exposures—for injury to manifest [47].

### NF1 Physiology and Vascular/Cardiac Abnormalities

Vascular complications are common in NF1, with systemic hypertension affecting 15% to 20%. Other CHDs include septal defects, aortic coarctation, and mitral anomalies [71, 72].

The NF1-HCM link is unclear; while animal models show cardiac hypertrophy with NF1 loss, human data are inconsistent [73–75]. Additional cardiac findings include mitral valve prolapse and rare arrhythmias [73, 76]. Emerging echocardiographic tools like speckle-tracking reveal subclinical dysfunction (e.g., reduced global longitudinal strain) despite preserved ejection fraction [77].

Cardiac risk in NF1 is heterogeneous. While conventional echocardiography may appear normal, some subgroups—those with large NF1 deletions or specific nontruncating mutations—show increased CHD risk, particularly pulmonary stenosis [72, 78]. Hypertension, often linked to renal artery stenosis, further elevates cardiovascular risk [71].

Routine cardiac screening is not universally recommended in NF1 patients, but baseline and follow-up assessments are prudent for high-risk patients—those with symptoms or genetic risk factors, or those receiving previous or concomitant cardiotoxic treatments [71, 79].

## Current Guidance and Guidelines

### Differences in the Package Inserts of Approved Medications

Prescribing information for MEKi consistently emphasizes cardiac monitoring, primarily focused on assessing LVEF, to mitigate risks of treatment-related cardiac dysfunction. Each product label recommends a baseline echocardiogram, with subsequent evaluations at varying intervals based on trial data and post-marketing experience (Table 3).

**Table 3:** Cardiac monitoring and dose modification guidance for MEKi (advice per package inserts)

MEKi	Recommended monitoring schedule	Hold/dose‑modification trigger (absolute ↓ in LVEF and value < LLN unless otherwise noted)	Resume/permanent discontinuation guidance	Key supporting data/label notes
Binimetinib	Baseline; 1 month; every 2–3 months	• >10% drop and < LLN (asymptomatic) • >20% drop and < LLN or symptomatic CHF	Withhold ≤4 weeks; resume at lower dose once LVEF recovers Permanently discontinue if unable to tolerate 30 mg BID	Product label guidance
Cobimetinib	Baseline; weeks 5, 17, and 29; every 4–6 months; extra check 2–16 weeks post‑resumption	• ≥10% drop and < LLN (asymptomatic) • Symptomatic or sustained ≥1% drop and < LLN	Withhold 2 weeks; resume at reduced dose if resolved If decline persists ≥4 weeks or symptomatic, resume at reduced dose or discontinue	Used with vemurafenib; monitoring per label
Mirdametinib	Baseline; every 3 months (year 1); thereafter PRN	• ≥10% drop and < LLN (hold; may resume ↓ dose on recovery) • ≥20% drop from baseline (hold regardless of LLN)	Resume at reduced dose once recovered Permanently discontinue if ≥20% decline persists	ReNeu trial: Asymptomatic (treatment-related) LVEF declines—12% in adult patients, 20% in pediatric patients
Selumetinib	Baseline; every 3 months (year 1); every 6 months thereafter	• ≥10% drop and < LLN (hold) • Grade 3/4 or symptomatic decline	Resume at reduced dose once recovered Discontinue for grade 3/4 or symptomatic events	Pediatric NF1 data: ~23% asymptomatic cardiomyopathy
Trametinib	Baseline; 1 month; every 2–3 months	• Asymptomatic ≥10% drop and < LLN (hold) • >20% drop and < LLN or symptomatic (hold)	Withhold ≤4 weeks; resume at reduced dose if LVEF normalizes Permanently discontinue for symptomatic or severe declines	METRIC: decreased ejection fraction/ventricular dysfunction—7% in adult patients COMBIv: asymptomatic LVEF decline—8% in adult patients, N/R in pediatric patients COMBId: LVEF decline—4% in adult patients; N/R in pediatric patients

BID, twice daily; CHF, congestive heart failure; LLN, lower limit of normal; LVEF, left ventricular ejection fraction; MEKi, mitogen-activated protein kinase kinase inhibitor; PRN, as needed; N/R, not reported

Collectively, these labels offer similar caution about cardiac dysfunction, focused on a view of CTCAE v.5.0–defined AEs. This expert panel agrees that changes in ejection fraction were an important feature to study during drug safety evaluations. Although the exact monitoring frequencies vary, the threshold for intervention—typically an absolute decline of ≥ 10% with a value below the lower limit of normal—remains consistent. Permanent discontinuation has been reserved for symptomatic cardiomyopathy or more profound LVEF reductions that do not resolve with drug holds, particularly in patients prescribed combined MEK and BRAF inhibitors. In the sections that follow, we explore the risks of developing heart failure and how clinical surveillance may be safely adapted to improve consistency in medical administration, reduce complexity of surveillance, increase access to these medications, and ultimately improve overall patient outcomes.

### Pharmacovigilance Findings and the Development of Monitoring Strategies

The cardiac monitoring strategies for MEKi therapy are primarily informed by prospective safety data from pivotal registration trials and are codified in product labels. Across agents, these trials implemented routine echocardiographic surveillance to detect both symptomatic and asymptomatic declines in LVEF, with coding of AEs according to the CTCAE, which guided subsequent dose modification or discontinuation thresholds (Table 3).

In the coBRIM trial, which supported the approval of cobimetinib in combination with the BRAF inhibitor vemurafenib, echocardiograms were performed at baseline and at fixed intervals (weeks 5, 17, 29, and 43 and every 4–6 months thereafter). LVEF decline was observed in 26% of patients, leading to dose modification in 22% and permanent discontinuation in 14%, with resolution in 62% of cases following intervention. These data shaped the frequency and clinical thresholds for monitoring embedded in the label [80]. Importantly, the incidence of reduced ejection fraction was higher with cobimetinib plus vemurafenib (8%) compared to the BRAF inhibitor alone (3%), highlighting the additive cardiac impact of MEK inhibition. Similarly, the COMBI-d program reported an 8% incidence of LVEF decline with BRAF inhibitor dabrafenib plus MEKi trametinib but none with vemurafenib alone, reinforcing the role of MEKi in these cardiac effects [57, 58]. The extended 5‑year follow‑up of coBRIM reinforced this signal: protocol‑defined grade ≥ 2 ejection fraction reduction occurred in 13% of combination‑treated patients versus 4% with control (vemurafenib + placebo), LVEF decline prompted cobimetinib discontinuation in 2%, and fatal cardiac events (cardiac arrest or myocardial infarction) were reported in 2% of patients receiving the dual‑targeted regimen [55].

MEKi monotherapy trials have adopted similarly intensive cardiac surveillance despite usually milder dosing regimens and known lower risks associated with MEKi alone compared with MEKi + BRAF inhibition, underscoring the class‑wide approach to proactively monitor LVEF.

The ReNeu trial of mirdametinib in patients with NF1 and symptomatic, inoperable PN incorporated serial cardiac assessments, revealing ≥ 10% LVEF decline in 12% of adults and 20% of children (treatment-related), most within the first 4 months of treatment. Most of these declines in heart function were still within the normal range, or into the borderline or mildly depressed range. Echocardiographic monitoring every 3 months during the first year of therapy has become the package insert recommendation based on these findings, as most declines were reversible (75%) and all were asymptomatic [15, 81].

In the SPRINT Phase 2 trial of selumetinib in pediatric NF1-PN, asymptomatic LVEF declines were detected in 7 of 50 children (14%). Echocardiography was scheduled every 4 months through month 25 (~ 2 years) and every 6 months thereafter—one of the longest routine cardiac monitoring schedules among approved MEKi [14, 82]. The US prescribing information, now reflecting a pooled pediatric NF1‑PN safety population (*N* = 134), reports grade 2 LVEF decrease (40%–50%; 10–19% drop from baseline) in 17% of evaluable patients, with decreased LVEF resolving in 75% [83].

For trametinib, the METRIC, COMBI-d, and COMBI-v trials formed the basis of monitoring recommendations. These trials incorporated routine LVEF assessments and identified cardiomyopathy rates between 6% and 9%. Consequently, the prescribing information advises baseline LVEF evaluation followed by assessments at 1 month and every 2 to 3 months thereafter [29, 50, 57, 84].

Robison et al. published their experience with predominantly trametinib-based pediatric therapy and demonstrated that ejection fraction declines were relatively common but reversible, mild, asymptomatic, and, while they mostly resolved off therapy, they also frequently improved spontaneously during ongoing therapy.

Overall, the trial-derived data substantiates the common occurrence of small decreases in LVEF, but cases of clinically significant declines in cardiac function resulting in clinical heart failure are extremely rare. Cases of heart failure have occurred in patients with other potentially recognizable risk factors, including chronic inflammatory conditions such as systemic lupus erythematosus, or in the setting of pre-existing chronic cardiovascular disease. Given that the number of dose reductions and drug interruptions is very large in these studies, potentially limiting the therapeutic benefit of the therapy itself, we believe that the combination of (1) shifting from imaging-based to biomarker-based surveillance and (2) taking a “permissive cardiotoxicity” approach to MEKi therapy can benefit the treatment populations as a whole.

### Overview and Limitations of Current Guidelines

Current labeling guidelines mirror clinical trials with a substantial emphasis on cardiac imaging. However, cardiac biomarkers may be more sensitive to cardiomyocyte damage (troponin or cardiac CK-MB) or important cardiac physiological stress (BNP or NT-proBNP) than cardiac imaging, without the issues related to expense or access to care that accompany echocardiography. Studies citied above emphasize changes in cardiac ejection fraction, which are nonspecifically related to many other factors of health and disease, such as the stress of early disease, hypertension, cardiac energetics, and many cellular pathways affected by the MEK-BRAF-ERK pathway, which influence cardiac sarcomeric contractility without influencing cell health.

The field of cardio-oncology has matured significantly over the past 2 decades, marked most recently by the publication of comprehensive guidelines from the European Society of Cardiology (ESC) in 2023 and consensus recommendations from the International Cardio-Oncology Society (IC-OS) in 2020. These guidelines have not yet been adapted to the relatively new experience and uses of MEKi, particularly as monotherapy (Table 1).

Current guidelines focus on reduction of dosing or cessation of therapy in response to changes in cardiac ejection, without clear evidence that minor changes in cardiac ejection are important to short- or long-term patient outcomes. This defensive cardiac stance leads to many therapy changes and interruptions, which impede a patient’s ability to benefit from therapy. We would like to introduce to this paradigm what cardio-oncologists allude to as “permissive cardiotoxicity,” or permitting some amount of abnormality in cardiac function or biomarkers to occur without change in therapy [85]. Permissive cardiotoxicity acknowledges the importance of both the expected endpoint of a patient’s disease and the acceptable limits of reversible or permanent cardiac decline. The concept is similar in some ways to the use of renal toxic antibiotics, where known toxicity is accepted based on the importance of the therapy.

International guidelines remain focused on combination RAF-MEK inhibitor regimens, such as those used in *BRAF*-mutant melanoma. As a result, the nuanced surveillance needs of pediatric and NF1 populations treated with MEKi monotherapy remain insufficiently addressed [86–91].

In practice, the most common issue of small, asymptomatic changes to ejection fraction poses little risk of progression to clinical heart failure, and it also prioritizes possibly irrelevant cardiac measurements over the benefits of the medication therapy, creating a risk for clinicians and patients when therapy is too often interrupted or reduced.

### Surveillance

**Fig. 2:**
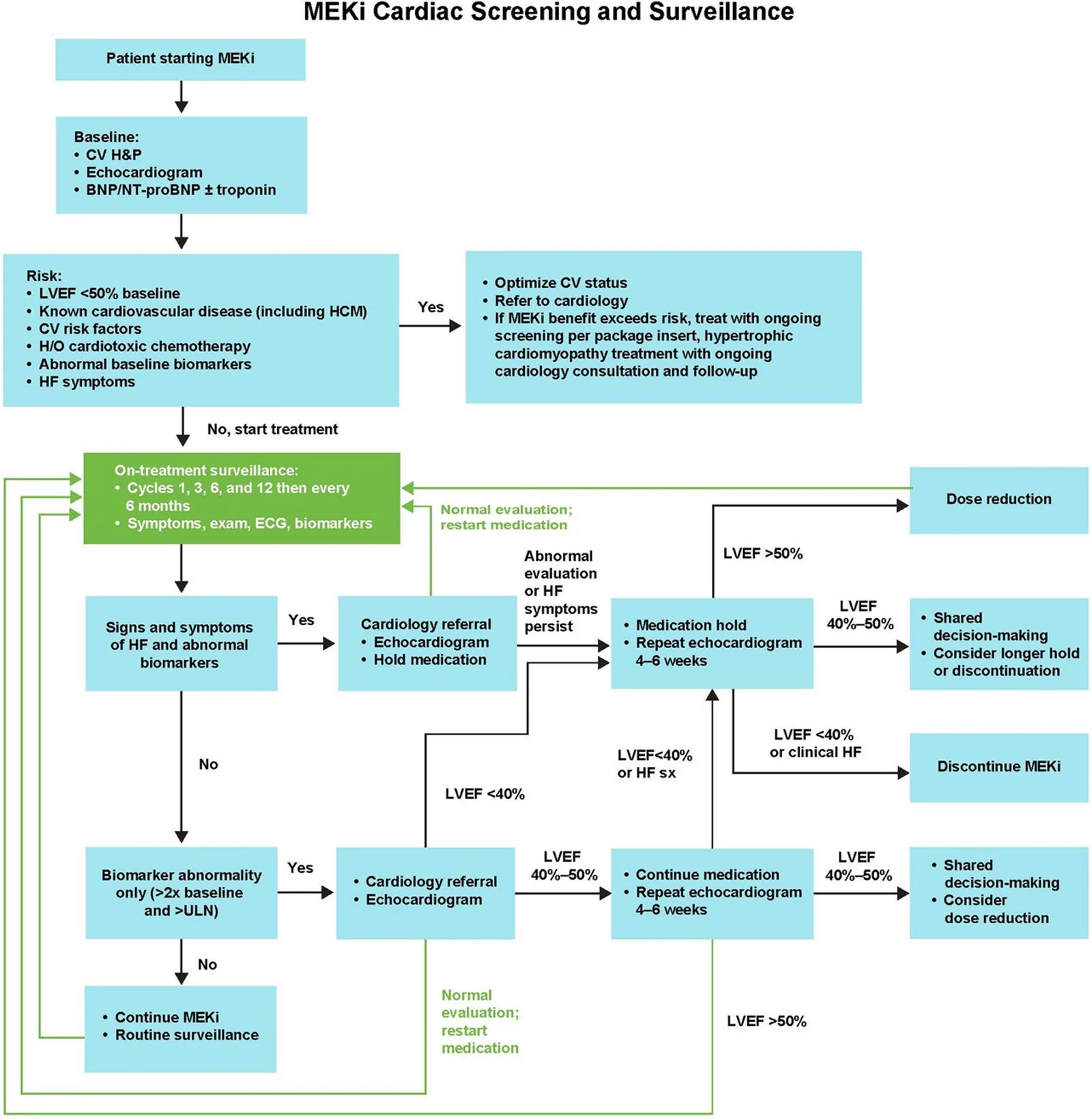
MEKi Cardiac Screening and Surveillance. This algorithm can be used to determine the correct approach to cardiac screening and surveillance in patients initiating MEKi therapy. Initial risk assessment and biomarker screening (BNP/NT-proBNP, troponin) guide intensity of follow-up. Echocardiography and cardiology referrals are reserved for high-risk patients and those with significant biomarker elevations or symptomatic cardiac changes. BNP, B-type natriuretic peptide; CV, cardiovascular; ECG, echocardiogram; HCM, hypertrophic cardiomyopathy; HF, heart failure; LVEF, left ventricular ejection fraction; MEKi, mitogen-activated protein kinase kinase inhibitor; NT-proBNP, N-terminal pro–B-type natriuretic peptide; sx, symptoms

Cardiac biomarkers, particularly BNP or NT-proBNP and cTn, are a viable alternative and adjunct to imaging-based surveillance. These markers signal early myocardial stress or injury and have proven useful in detecting cardiotoxicity in patients receiving anthracyclines and immune checkpoint inhibitors (ICIs). Substantial changes in these biomarkers can signal clinically relevant changes in cardiovascular health and can serve as a sensitive screening mechanism to determine when expensive and less available imaging may be relevant or required. ESC guidelines recommend routine serial measurements of these biomarkers in patients receiving agents such as vascular endothelial growth factor inhibitors, bortezomib, and HER2-targeted therapies; analogous thresholds for MEKi therapy are outlined in Fig. 2.

In selected contexts, biomarkers may serve as a viable component of rationalized surveillance. Elevated pretreatment biomarkers may help identify patients who warrant closer imaging surveillance. For example, BNP monitoring is advised every cycle for the first 6 cycles of the proteasome inhibitor bortezomib (used for treating multiple myeloma, among other conditions), even in normal-risk patients—a recommendation made based on general experience rather than specific bortezomib-related evidence. Similarly, troponin screening in ICI-treated patients has excellent sensitivity for myocarditis cases but is complicated by high false-positive rates and unclear downstream management implications [90, 92]. In patients with persistently normal biomarkers beyond the initial months of therapy, particularly in lower-risk settings, imaging frequency could reasonably be reduced. Cardiac biomarker measurements are both available (stable in serum after phlebotomy to be measured in nearly any outpatient setting) and relatively inexpensive (combinations of BNP and troponin are generally < 5% of the cost of an echocardiogram), contrary to many healthcare environments where imaging capacity is constrained or even unavailable. In this way, biomarkers offer a practical, lower-cost tool that can facilitate broader implementation of both disease-related therapy and cardio-oncology (heart failure) therapies for appropriate patients. While biomarkers are unlikely to fully replace imaging in all contexts, they offer a promising avenue to enhance the efficiency, personalization, and accessibility of surveillance strategies. Future studies are essential to define optimal thresholds, longitudinal trajectories, and clinical algorithms to improve the specificity and utility of these tools in cardio-oncology care.

### Recommendations for Baseline Screenings and Ongoing Surveillance

This panel sought to evaluate the current screening recommendations for MEKi monotherapy to decide whether the individual components of the recommended screening were important to treatment initiation, treatment success, and avoidance of significant toxicities. We are limited substantially by product labeling that suggests and relies upon frequent echocardiography rather than biomarker monitoring, perpetuations of screening behaviors initiated as part of pharmacovigilance in Phase 2 and 3 clinical trials. Based on clinical experience, we pose the following incremental changes to cardiac surveillance for MEKi monotherapy in a variety of pediatric and adult indications:

1. Identification of potential risk factors for cardiac sensitivity to MEKi therapy. A set of recommended baseline studies that provide adequate screening for risk factors that might increase risk of cardiac morbidity during MEKi therapy (Tables 4 and 5).
  Patients without such risk factors appear to be at very low risk of substantial cardiac dysfunction attributable to MEKi therapy.
2. A pathway for cardiac screening and ongoing assessment of at-risk individuals, which includes the involvement of a cardiologist and echocardiographic surveillance adhering to the product labels (Table 3).
3. An amended structure for cardiac surveillance of the remaining, normal-risk individuals, stressing the identification of important cardiac physiological changes over the identification of minor, asymptomatic, and potentially fleeting changes in cardiac contractility (Table 4).

**Table 4:** Normal‑risk surveillance checklist for MEKi monotherapy

Component	Details
Timing	Cycle 1 (4 weeks); Cycle 3 (12 weeks); Cycle 6 (24 weeks); Cycle 12 (48 weeks); then every 6 months
Symptom review	• Dyspnea on exertion/↓ exercise tolerance • Palpitations or angina • Syncope or orthostatic symptoms • Orthopnea or PND
Vital signs	• Resting pulse (new tachycardia) • Blood pressure changes (hypertension/hypotension) • Respiratory rate elevation • Oxygen saturation decline • Unexplained weight gain (fluid retention)
Physical examination	• Cardiovascular: rhythm, murmurs, pulse strength, edema, perfusion • Respiratory: crackles, wheeze, ↑ work of breathing • Abdominal: hepatomegaly, ascites • Dermatologic: MEKi‑associated rashes
Laboratory investigations	• Comprehensive chemistries (renal panel, liver transaminases, bilirubin) • Complete blood count with differential • Cardiac biomarkers: troponin T/I (or CK‑MB if not available) and BNP or NT‑proBNP

MEKi, mitogen-activated protein kinase kinase inhibitor; PND, paroxysmal nocturnal dyspnea

Our hope is that with this slightly reduced intensity of echocardiographic surveillance, more providers will be able to provide screening and surveillance for patients requiring MEKi therapy, in a broader range of clinical settings. Further, we hope that this strategy may reduce dose interruptions significantly but safely, leading to greater likelihood of treatment success without significant increase in adverse outcomes.

### Screening Potential Patients

Because there are multiple indications for MEKi therapy, the screening recommendations must also be tailored to the drug indication. Further, because the specialists prescribing MEKi include cardiologists, neurologists, and oncologists, in addition to general practitioners in some settings, we include relatively specific elements for assessment during a history and physical examination. The following list must be viewed as complementary to disease-specific biomarkers and imaging (Table 4). Further, because MEKi have potential side effects not related to the cardiovascular system, including, but not restricted to, cutaneous and ophthalmologic effects, these baseline studies must be viewed as complementary to other modes of screening such as skin and eye examination.

1. Patient history with attention to chronic disease identification and family history of cardiac disease/events.
  - Review of symptoms: dyspnea on exertion, palpitations, angina, syncope, orthostasis.
  - Personal history: hypertension, coronary disease/myocardial infarction, hyperlipidemia, rheumatologic or chronic inflammatory disease (particularly incompletely controlled diseases, including lupus, rheumatoid arthritis, etc.), and other vital organ disorders (renal, pulmonary, hepatic, etc.) as they pertain to increased patient sensitivity to changes in cardiac function.
  - Family history of cardiovascular disease, cardiomyopathy, myocardial infarction or heart failure before age 50, systemic inflammatory disease.
2. Vital signs: pulse, blood pressure, respiratory rate, oxygen saturation.
3. Physical examination: nutrition (cachexia, obesity), cardiovascular exam (heart rate and rhythm, murmurs, pulse strength, edema, perfusion), respiratory exam (abnormal sounds, inspiratory effort or rales, expiratory prolongation/wheezing), abdominal exam, liver enlargement or ascites.
4. Baseline laboratory testing.
  - Comprehensive chemistries with renal function, liver transaminase level, bilirubin.
  - Complete blood counts with differential.
  - Cardiac biomarkers: either troponin T or troponin I (optionally high sensitivity assay; if unavailable, CK with MB fraction) and BNP or NT-proBNP.
5. Baseline echocardiogram with assessment of left ventricular systolic and diastolic function.
6. Baseline electrocardiogram (ECG) to assess for conduction abnormalities or baseline significant prolongation of QT interval.

### Risk Factors and Thresholds for Cardiology Referral

The risk of cardiovascular complications from cancer therapy is strongly influenced by baseline patient characteristics (Table 3). It is reasonable to consider such risk factors as plausible modifiers of risk for heart failure related to MEKi therapy, but given the low frequency of clinically significant cardiac events with these drugs, it will take time to define their role. The goal of the above baseline evaluation is to identify patients with significant concerning abnormalities who would benefit from the oversight of a cardiologist. It is noteworthy that many cardiologists may not be aware of MEKi therapy or the potential cardiac effects, so they may be less comfortable with so-called “permissive cardiotoxicity” approaches. Perhaps appropriately, therefore, cardiology involvement and repeat ECGs lend the possibility of treatment interruptions due to changes in cardiac performance.

**For patients with significant concerning abnormalities on the baseline screening**,** we suggest adhering to package inserts for MEKi drugs regarding monitoring and therapeutic adjustments.**

The 2020 ESC position paper, developed in partnership with IC-OS [89], provides a structured framework for identifying individuals at heightened risk of treatment-related cardiotoxicity when undergoing traditional chemotherapy. Key risk factors include established cardiovascular disease—such as heart failure, coronary artery disease, arrhythmias, or valvular pathology—as well as common medical comorbidities like hypertension, diabetes mellitus, dyslipidemia, and chronic kidney disease. Lifestyle-related contributors, including smoking, obesity, sedentary behavior, and poor diet, further exacerbate cardiovascular vulnerability and may impede myocardial recovery following injury. In addition, baseline abnormalities in cardiac biomarkers, such as elevated cTn or BNP levels, although not fully validated for routine risk stratification, are increasingly viewed as indicators of subclinical cardiac disease and are being incorporated into initial risk assessments. Historical exposure to cardiotoxic therapies—such as anthracyclines, HER2-targeted agents, or thoracic radiation—also amplifies susceptibility and should inform surveillance intensity [89]. However, dedicated studies to delineate specific risk predictors for MEKi-associated cardiac effects are lacking and represent an area of unmet research need.

To support clinical decision-making, the ESC guidelines include a referral algorithm that outlines when patients undergoing cancer therapy should be evaluated by cardiology. While the risk stratification framework informs this process, the referral criteria themselves are not prescriptive and must be interpreted in the context of the individual patient’s cumulative risk profile. Accordingly, for patients initiating or receiving MEKi therapy, it is reasonable to consider cardiology referral in those with ESC-acknowledged risk factors (Box 1).

**Box 1.** ESC-Acknowledged Risk Factors That Should Prompt Cardiology Referral Prior to MEKi Therapy

**Table:** 

• Established cardiovascular disease or prior history of decreased heart function or heart failure (for pediatric patients, structural cardiac abnormalities or hypertrophic cardiomyopathy as detected on baseline echocardiogram are included) • Lifestyle risk factors (e.g., smoking, sedentary lifestyle, poor diet, obesity) • Prior treatment with cardiotoxic medications, such as anthracycline agents • Baseline abnormalities of cardiac biomarkers (troponin, CK-MB, BNP, NT-proBNP) • Baseline symptoms potentially attributable to cardiovascular disease, such as dyspnea on exertion, orthopnea, syncope, or palpitations (for pediatric patients, edema, hepatomegaly, pleural effusion, or ascites in the setting of RASopathy are included)	

The following criteria outline important clinical triggers for cardiology evaluation in patients undergoing MEKi monotherapy. Prompt cardiology assessment in response to these indicators can facilitate timely intervention and management of potential cardiac AEs, ensuring patient safety and treatment continuity (Box 2).

**Box 2.** Triggers for Cardiology Evaluation During MEKi Monotherapy

**Table:** 

• Development of new cardiovascular symptoms, such as dyspnea on exertion or orthopnea • New electrocardiographic abnormalities, such as atrial fibrillation or QTc prolongation, especially paired with syncope or palpitations • Greater than doubling of baseline BNP or NT-proBNP, exceeding the upper limit of normal • New abnormal elevation of troponin or CK-MB • Mild, asymptomatic reductions in LVEF to approximately 40%–50% without biomarker elevation or heart failure signs **need not** automatically interrupt MEK therapy; treatment may continue with cardiology input and repeat biomarkers ± echocardiogram in 4–6 weeks, reserving dose modification for progressive ejection fraction decline or overt symptoms (a permissive cardiotoxicity approach)	

### Surveillance for Normal-Risk Patients (Not Meeting Above Criteria)

Our suggestions for surveillance of patients not meeting the above criteria for cardiology evaluation include a reduced dependence on echocardiography and an increased utilization of clinical surveillance and biomarker data (Table 4).

Echocardiography: We note that not only are small reductions in cardiac ejection potentially unimportant, utilizing echocardiography at a reduced frequency not for clinical cause is likely to lead to idiosyncratic discovery of mild or borderline cardiac abnormalities in patients otherwise tolerating their drug well. Therefore, for patients with normal baseline echocardiogram, persistently normal biomarkers, and no clinical symptoms or signs of cardiac compromise, follow-up echocardiography is **not** recommended unless clinically indicated.

## Summary of Recommendations

Prior to MEKi therapy, patients should have a comprehensive history and physical focusing on potential threats to cardiovascular health. They should also have baseline cardiac studies, including an echocardiogram and ECG.

A baseline 12‑lead ECG should record rhythm, conduction intervals, and QTc to flag any pre‑existing abnormalities before the first MEKi dose. Follow‑up ECGs should be performed if new symptoms or biomarker abnormalities occur to inform cardiology input.

Patients with significant concerning abnormalities should be referred to cardiology for consultation and follow-up during therapy.

Patients without such abnormalities can be referred to as “normal-risk” patients, and we suggest a follow-up schedule with visits, history, physical, and blood testing, including cardiac biomarkers, after months 1, 3, 6, and 12 and every 6 months thereafter.

Patients with significant changes to biomarkers or end organ function should be referred for echocardiogram and/or cardiology consultation, with consideration of dose reduction or interruption (Tables 4 and 5).

**Table 5:** Practical recommendations for cardiac monitoring and dose adjustment during MEKi therapy

Aspect	Concise recommendation
Baseline work‑up	CV history/exam, ECG, echo, biomarkers (BNP or NT‑proBNP, troponin)
Surveillance schedule	Cycles 1, 3, 6, and 12; then q 6 mos: symptoms, exam, biomarkers
Echo frequency	Baseline compulsory; thereafter only if high risk or prior ≥10 pp LVEF drop and/or < LLN
Hold therapy	LVEF ≥10 pp drop < LLN + HF symptoms, severe arrhythmia/ACS, or persistent biomarker surge
Dose reduction	Symptomatic HF resolving ≤4 weeks or recurrent asymptomatic LVEF drop below LLN
Repeat testing	Abnormal biomarkers → repeat 1–2 weeks; confirm LVEF drop with echo ≤4 weeks
Cardiology referral	Baseline risk factors identified, LVEF <50%, symptomatic HF/arrhythmia, refractory biomarkers, diagnostic uncertainty
Managing EF change	No action for asymptomatic mild LVEF dip ≤10 pp or within normal range; don’t re‑escalate failed dose

ACS, acute coronary syndrome; CV, cardiovascular; ECG, electrocardiogram; EF, ejection fraction; FDA, US Food and Drug Administration; HF, heart failure; LLN, lower limit of normal; LVEF, left ventricular ejection fraction; MEKi, mitogen-activated protein kinase kinase inhibitor; q 3 mos, every 3 months; q 6 mos, every 6 months; yr, year

**Box 3.** On‑Label and Off‑Label Uses of MEKi

**Table:** 

• MEKi now treat diverse RAS/RAF/MAPK‑driven cancers beyond their original narrow labels • Selumetinib and mirdametinib are FDA approved for treatment of inoperable or symptomatic PN associated with NF1 • Trametinib is approved as monotherapy for *BRAF* V600E/K-mutant unresectable or metastatic melanoma • Cobimetinib is approved as monotherapy for histiocytic neoplasms • Approvals remain mutation‑ and histology‑specific, but trials are testing biomarker‑guided combinations • All use of MEKi for Noonan syndrome and other RASopathies is currently off-label • Trametinib leads use; binimetinib, cobimetinib, selumetinib, and mirdametinib are rising in off‑label regimens to overcome resistance • Growing off‑label data shift therapy toward pathway‑driven precision oncology, with future approvals likely based on molecular profiling (Table 1)	

### Knowledge Gaps

Despite the clinical use of MEKi for NF1-PN, gaps in clinical knowledge remain. For one, it is unclear if the prognosis for ejection fraction/shortening fraction declines when therapy continues without dose reduction or interruption; preliminary National Cancer Institute data suggest treatment interruptions (distinct from dose reductions, which may preserve therapeutic benefit) may raise progression risk [14, 88, 93, 94]. Second, there is a lack of evidence supporting specific cardiac monitoring schedules (e.g., echocardiogram cadence, biomarker triggers) during MEKi therapy [94]. Third, the effects of dose reduction on efficacy are mixed in melanoma treated with BRAF + MEK inhibitors, warranting additional comparative studies—clinical trials of planned intermittent dosing suggest reduced progression-free survival compared with continuous dosing, while real-world observational data indicate frequent dose reductions or interruptions associated with apparently lower efficacy, although confounding factors complicate interpretation [93, 95, 96]. The differences between adult and pediatric patients remain under‑characterized: baseline variability, frequency, and reversibility of ejection fraction/shortening fraction drops—and their clinical relevance—are uncertain [88, 94, 97]. Finally, the long‑term impacts of chronic MEKi use on growth, myocardial/vascular health, and other toxicities are poorly defined, though potential vascular benefits (e.g., NF1 vasculopathy improvement) merit investigation (Table 1) [92, 94, 98].

## Conclusion

After a decade of careful pharmacovigilance study regarding the cardiovascular effects of MEKi therapy, these agents have proven to be quite safe. Small, asymptomatic LVEF dips of approximately 10% points even to below the normal range (e.g., 45%–50%) are usually benign, and mild cardiac dysfunction with LVEF 40% to 45% may spontaneously improve while patients are receiving therapy. Interruptions or dose changes should be reserved for instances of signs or symptoms of heart failure, significant biomarker abnormalities, or LVEF well below normal limits (LVEF < 40%). Our proposed screening and surveillance protocol minimizes dependence on expensive and inaccessible cardiac echocardiography and maximizes the potential for interruption-free treatment with these beneficial medications. Ultimately, individualized care—balancing product labels, clinical judgment, and multidisciplinary input—is essential for optimizing outcomes in patients receiving MEKi monotherapy.

## Key References

- Moertel CL, Hirbe AC, Shuhaiber HH, et al. ReNeu: a pivotal, phase IIb trial of mirdametinib in adults and children with symptomatic neurofibromatosis type 1-associated plexiform neurofibroma. J Clin Oncol. 2025;43(6):716–29. (of outstanding importance)
  - ⚬ This phase 2b study measured the tumor response and safety of mirdametinib in adults and children with NF1-PN. Mirdametinib demonstrated durable tumor responses and improvements in pain and QoL in study participants. No adults or children had a symptomatic ejection fraction decrease. Mirdametinib is approved for the treatment of PN in patients with NF1.
- Gross AM, Wolters PL, Dombi E, et al. Selumetinib in children with inoperable plexiform neurofibromas. N Engl J Med. 2020;382(15):1430–42
  - ⚬ This open-label phase 2 clinical trial measured the safety and efficacy of selumetinib in pediatric patients with NF1-PN. Most enrolled patients had durable tumor shrinkage and showed clinical benefit from selumetinib after one year of treatment. No patients in the trial had symptomatic changes in left ventricular ejection fraction. Selumetinib is approved for the treatment of PN in patients with NF1.
- Glen C, Adam S, McDowell K, et al. Cardiotoxicity of BRAF/MEK inhibitors: a longitudinal study incorporating contemporary definitions and risk scores. JACC CardioOncol. 2023;5(5):628–37. https://doi.org/10.1016/j.jaccao.2023.04.004. (of importance).
  - ⚬ This is a longitudinal study in patients with melanoma to investigate the incidence, risk factors, and time course for treatment-related cardiac dysfunction in patients taking combination BRAF/MEKi therapy.
- Glen C, Tan YY, Waterston A, et al. Mechanistic and clinical overview cardiovascular toxicity of BRAF and MEK inhibitors: JACC: CardioOncology State-of-the-Art Review. JACC CardioOncol. 2022;4(1):1–18. https://doi.org/10.1016/j.jaccao.2022.01.096. (of importance).
  - ⚬ This comprehensive review summarizes data from multiple studies to describe the mechanisms of MEKi-associated cardiac toxicity.

## Data Availability

No datasets were generated or analysed during the current study.
